# Supplementary material for: Influence of the Surface Chemistry of Graphene Oxide on the Structure–Property Relationship of Waterborne Poly(urethane urea) Adhesive
Source: Materials (Basel). 2021 Aug 5;14(16):4377. doi: 10.3390/ma14164377 (PMC8399831; doi:10.3390/ma14164377)
Supplement: Supplementary file 1 [file materials-14-04377-s001.zip › materials-1305551-supplementary.pdf]

materials

MDPI

Influence Of The Surface Chemistry Of Graphene Oxide On The Structure-property Relationship Of Waterborne Poly(urethane urea) Adhesive

Abir Tounici, José Miguel Martín-Martínez

Supplementary Materials

Characterization of the graphene oxide derivatives

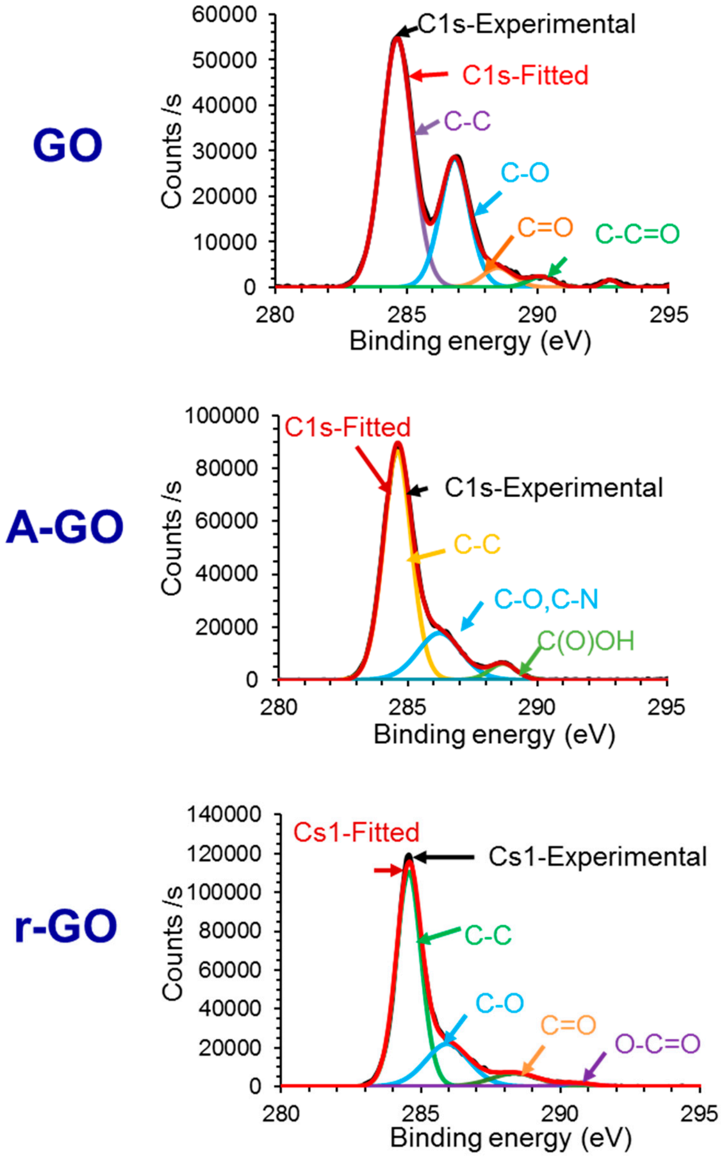

Figure S1. Curve fittings of the high resolution C1s spectra of the graphene oxide derivatives.

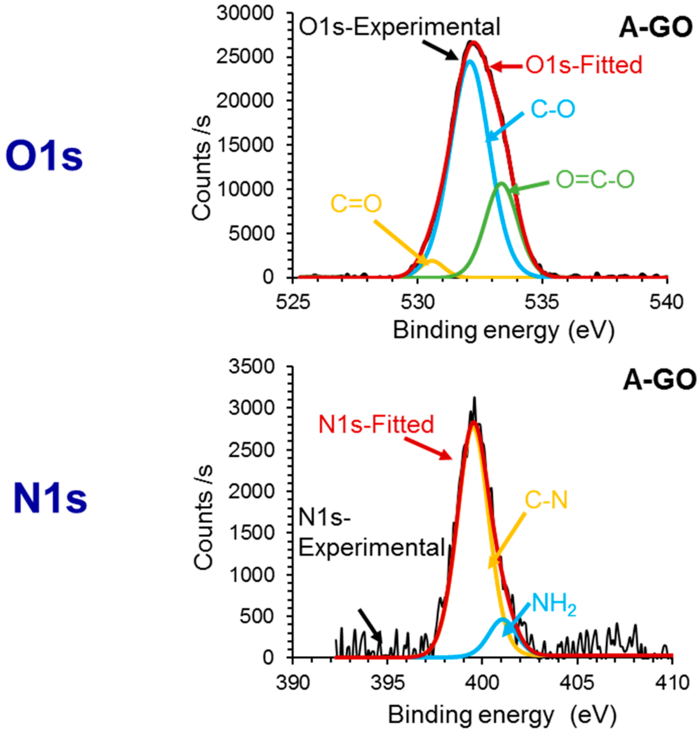

Figure S2. Curve fittings of the high resolution O1s and N1s spectra of A-GO.

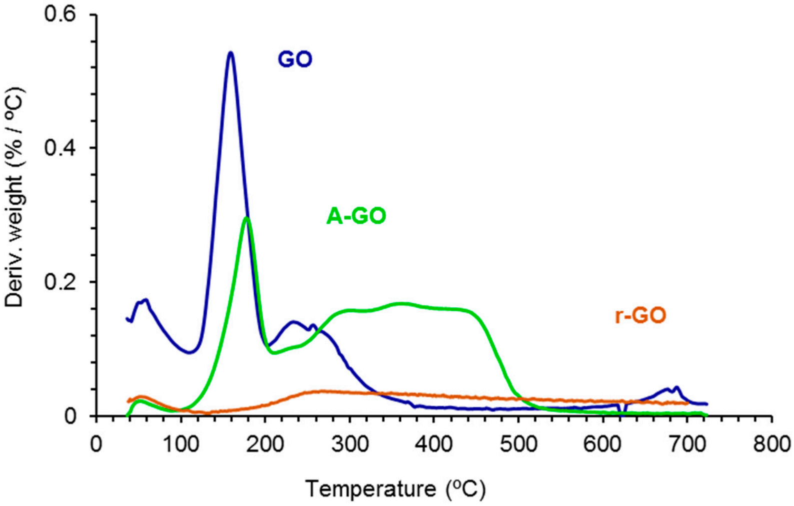

Figure S3. Variation of the derivative of the weight of the graphene oxide derivatives as a function of the temperature. TGA experiment.

Characterization of the poly(urethane urea)s (PUs)

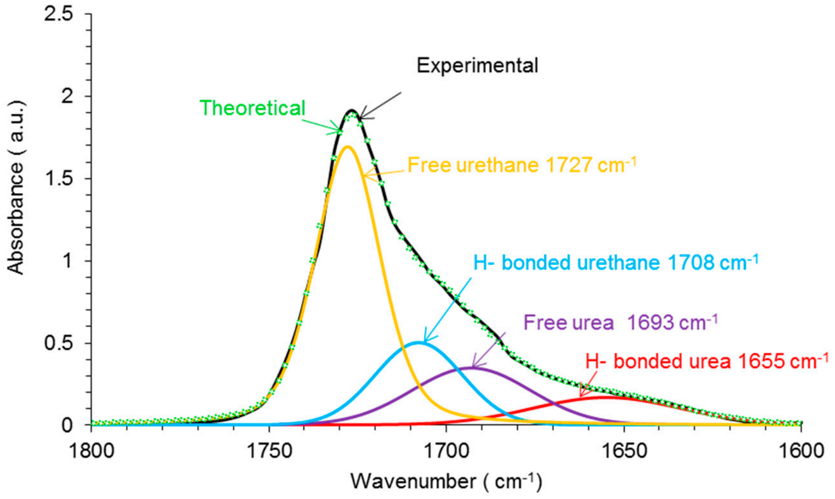

Figure S4. Curve fitting of the carbonyl region (1800-1600 cm^{-1}) of the ATR-IR spectrum of PU.

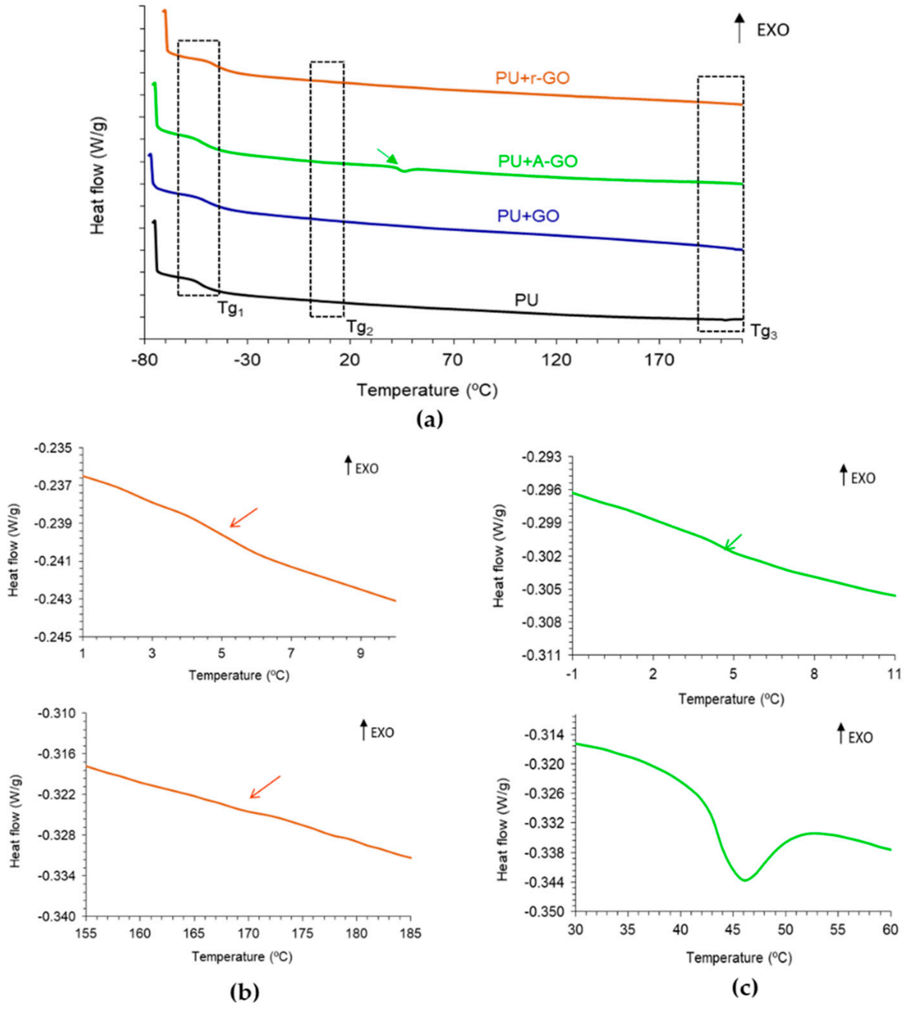

Figure S5. **(a)** DSC traces of the PUs. Second heating run; **(b)** Expanded regions of the DSC trace of PU+r-GO showing the glass transitions. Second heating run; **(c)** Expanded regions of the DSC trace of PU+A-GO showing the glass transition and the melting process. Second heating run.
